# Disruptors

**DOI:** 10.1093/jhps/hnaf015

**Published:** 2025-05-05

**Authors:** Richard E Field

**Affiliations:** City St George’s University of London

Few would be surprised if historians of the 22nd century describe 2025 as a year of heightened social, economic, and political disruption. It remains to be seen how these historians will judge the contribution of the individuals spearheading these changes [1–3]. In medical practice, disruptors face many challenges and checks that may divert or halt their progress. Alongside successful innovators such as Jenner (vaccination), Semmelweis (hand washing), Lister (antisepsis), Fleming (antibiotics), Charley (hip replacement), Murray (renal transplant), and the thousands of researchers developing cancer treatments, not a year passes without a would-be medical innovator being exposed for overconfidence [4], failing to satisfy regulatory or legal checks [5], falsifying results [6], or exploiting vulnerable individuals [7].

Thirty years ago, hip preservation surgeons were generally viewed by their peers as misguided disruptors. This attitude has changed and at the 2025 British Hip Society meeting large sections of the conference were devoted to the dissemination of hip preservation knowledge and practice [8].

One of the requirements for a disruptor’s innovation to be adopted by the wider community is that the innovation provides beneficial and reproducible outcomes. While interventions such as resection of a Cam bump or repair of a torn labrum may relieve pain and enable a patient to return to high function [9], there is growing awareness that the outcome of such interventions will also be affected by pre-existing degenerative changes [10], subtle variations in the geometry of the proximal femur and acetabulum [11], the age and sex of the patient [12], and the envelope of surrounding soft tissue structures [13].

In this issue, we present a paper from Akshar Thakkar *et al*. [14] who have investigated the 2-year rate of re-arthroscopy or progression to Total Hip Arthroplasty (THA) of 19 067 patients whose hip arthroscopies were recorded on the PearlDiver database between 1 October 2015 and 31 October 2018. The authors found that the overall, 2-year, reoperation rate was 18.58%. Of these, 11.42% had undergone re-arthroscopy of the same hip and 7.16% had undergone THA of the affected hip. These results are disturbing for several reasons. First, revision femoroplasty was recorded for 72% of the cases who underwent re-arthroscopy. This either indicates that an inadequate femoroplasty was undertaken at the index operation or that the true aetiology of the patients’ preoperative symptoms was poorly understood. Secondly, a re-arthroscopy rate of 11.42% only records patients who were willing to undergo a repeat of the operation that had already proven unsatisfactory. It is unlikely that all the 81.42% who had not submitted themselves to further surgery were satisfied with the outcome of their index procedure. In a time of increasing awareness of the need for surgical interventions to provide consistent clinical and health economic benefits, these figures indicate that some hip preservation surgeons could improve their indications for hip arthroscopy and the technical execution of their interventions.

A second requirement for adoption of a disruptive innovation is that it achieves better outcomes than alternative solutions. In clinical practice, a single outcome measure seldom provides clinicians with an instrument by which to decide which surgical intervention would best serve a particular patient. This challenge is addressed by Panayiotis Christofilopoulos’ team in their paper investigating muscle strength recovery following 6 months of rehabilitation for patients who have undergone surgical hip dislocation for femoroacetabular impingement [15]. The authors observe that, in cases with more severe or complex pathology, surgical hip dislocation may provide a better surgical correction than can be achieved through an arthroscopic intervention. However, the authors do identify a potential downside to this strategy as their patients failed to regain normal external hip rotator power by the 6-month timepoint. This contrasts with Leunig’s study [16] of muscle power after arthroscopic treatment of femoroacetabular impingement. While Leunig did not see this deficit, his study assessed patients at a later timepoint and did identify a persistent deficit in hip flexor power.

Many disruptive innovations will enjoy temporary popularity before being discarded because of unexpected consequences or economic disadvantages. The latter is particularly important for sustained adoption and the paper from the team at the Nicholas Institute of Sports Medicine and Athletic Trauma [17] demonstrates that, with appropriate supervision, professional athletes can return safely to competitive sporting activity more quickly than was previously recognized. However, such patients are among the fittest undergoing hip preservation surgery, and, in everyday practice, most surgeons may find it prudent to remind their patients that tissue healing continues at a molecular level for 15 months and that it may take a year or two to enjoy the full benefit of hip preservation surgery.

We hope that you are spared unwelcome disruption to your work and home life in 2025, that you enjoy all the papers in Issue 12.1, and that you find them helpful to develop your hip preservation practice.
